# Parental Perceptions and Actual Oral Health Status of Children in an Italian Paediatric Population in 2024: Findings from an Observational Study

**DOI:** 10.3390/children12091119

**Published:** 2025-08-25

**Authors:** Claudia Capurro, Giulia Romanelli, Giulia Telini, Virginia Casali, Maria Grazia Calevo, Martina Fragola, Nicola Laffi

**Affiliations:** 1 Paediatric Dentistry and Orthodontics Unit, IRCCS Istituto Giannina Gaslini, 16147 Genova, Italy; claudiacapurro@gaslini.org (C.C.); giuliatelini@gaslini.org (G.T.); virginiacasali@gaslini.org (V.C.); nicolalaffi@gaslini.org (N.L.); 2 Biostatistics Unit, Scientific Directorate, IRCCS Istituto Giannina Gaslini, 16147 Genova, Italy; mariagraziacalevo@gaslini.org (M.G.C.); martinafragola@gaslini.org (M.F.)

**Keywords:** oral health, quality of life, children, adolescents, parental perception, family impact, dental visit, dental examination

## Abstract

**Background/Objectives:** Oral health plays a crucial role in the physical, emotional, and social well-being of children. Data from 2019 indicate that oral diseases remain a major concern in the Italian paediatric population, affecting not only children’s health but also caregivers’ well-being. This study aimed to assess the importance attributed by Italian families to their children’s oral health and correlate parents’ perceptions with children’s actual oral health status. **Methods:** A total of 131 children aged 0–12 years, admitted to the IRCCS Giannina Gaslini Children’s Hospital (Genoa, Italy) for reasons other than dental problems, were enroled between 1 January and 31 December 2024. Parents completed validated questionnaires (ECOHIS or PCPQ + FIS) based on their child’s age, along with supplementary questions on socio-demographic background and dental history. Oral examination was performed to assess dmft/DMFT scores, the Index of Orthodontic Treatment Need (IOTN), and the Modified Gingival Index (MGI). **Results:** In younger children (0–5 years), oral health was generally good, but the presence of caries negatively impacted the family’s quality of life. Older children (6–12 years) showed higher rates of caries and gingival inflammation, affecting their daily functioning and emotional well-being. Poor oral health was more common among children of non-European backgrounds and those with lower parental education. Early dental visits, within the recommended 24 months of age, were rare. **Conclusions:** Despite clear international recommendations, early dental visits remain uncommon, and many children experience preventable oral health issues. These findings highlight the urgent need to improve caregiver education and public health strategies to promote early preventive dental care.

## 1. Introduction

Oral health plays a fundamental role in overall well-being, supporting daily functions such as eating, speaking, and breathing. Beyond physical health, it also contributes to psychological and social aspects, influencing self-esteem, emotional balance, and the ability to interact and work without pain or discomfort [1]. In the paediatric population, oral conditions can negatively affect not only children’s general health and development but also their families’ social, working, and emotional well-being [2,3].

In 2019, the prevalence of oral health issues in Italy highlighted the widespread burden of untreated caries, periodontal disease, and edentulism, affecting not only adults [4] but also the paediatric population. Specifically, the prevalence of untreated caries in deciduous teeth among children aged 1–9 years was 36.1%, while among children older than 5 years, it was 29.6% in permanent dentition. Additionally, 18.2% of individuals aged ≥ 15 years were affected by periodontal disease [5]. The severity of clinical conditions can significantly impact the quality of life of children and their families. According to the World Health Organisation (WHO), Quality of Life (QoL) reflects an individual’s perception of their position in life, shaped by the cultural and value systems they live in, and influenced by their goals, expectations, standards, and concerns. QoL is a meaningful indicator for evaluating overall health, both physical and mental, including oral health [6]. In children, improper diagnosis can lead to significant functional consequences, such as phonetic issues, malocclusion, masticatory dysfunction, and aesthetic concerns. However, all these factors can contribute to the breakdown of a child’s emotional and social well-being, ultimately affecting their overall quality of life.

Over the past decade, significant strides have been made in developing and validating tools to assess children’s oral health-related quality of life (OHRQoL). This has led to the current availability of several options for recording the OHRQoL, including the Child Oral-Health-Related Quality of Life (COHQoL), the Child Oral Health Impact Profile (COHIP), and the Child Oral Impacts on Daily Performances (Child-OIDPs) questionnaires [7].

The COHQoL is the most frequently used scale, which evaluates the adverse effects of oral disorders on children and their families. It contains the Child Perceptions Questionnaire (CPQ), the Parental-Caregiver Perceptions Questionnaire (P-CPQ), and the Family Impact Scale (FIS). In particular, the P-CPQ has proven reliability and validity for children aged 6–14 years, showing responsiveness in longitudinal studies, especially when administered alongside the FIS [8]. A cross-sectional study conducted between 2023 and 2024 by Rincon et al. evaluated Oral Health-Related Quality of Life in early adolescents using the Child Perceptions Questionnaire (CPQ). The findings revealed a significantly high impact on emotional and social well-being, as well as elevated anxiety levels. Notably, all 130 participants, with a mean age of 12.6 years (±1.06), were attending their first dental visit, proving the burden of delayed dental care [9]. Supporting these results, a 2023 study applied the PCPQ and Family Impact Scale (FIS) to assess the effects of malocclusion and caries on adolescents’ quality of life. The data confirmed a pronounced overall impact, particularly on social well-being aspects [10].

For preschool children, a specific questionnaire, the Early Childhood Oral Health Impact Scale questionnaire (ECOHIS), was developed based on the P-CPQ [11]. The Italian version of this questionnaire was validated by Contaldo et al. in 2020 [12] and has since been used as a tool in cross-sectional studies, including a recent one conducted in Italy that investigated the parents’ critical role in children’s health, finding a correlation between children’s oral health status and parents’ educational level [13].

This study aimed to assess the importance that the Italian population places on their children’s oral health in 2024. This evaluation was based on an objective clinical examination of the children’s oral health status and its comparison with parents’ perceptions using the ECOHIS and PCPQ + FIS questionnaires.

The secondary outcome was to assess how sociodemographic factors could relate to children’s dental experiences, including both the frequency of dental visits and the occurrence of oral diseases.

## 2. Materials and Methods

### 2.1. Ethical Considerations

This observational study was conducted at the IRCCS Giannina Gaslini Children’s Hospital in Genoa, Italy.

The study protocol was reviewed and approved by the Regional Ethics Committee before participant recruitment began (N. CET-Liguria: 294/2023-DB id 13233, 2 October 2023).

### 2.2. Study Design and Participants

The study population comprised 131 subjects.

The inclusion criteria required children to be between 0 and 12 years of age, born in Italy with Italian or foreign parents, and admitted consecutively to the Giannina Gaslini Children’s Hospital between 1 January 2024 and 31 December 2024.

The exclusion criteria were as follows: children older than 12 years of age; critically ill conditions, such as patients with severe diseases such as cancers, encephalopathies, and rare genetic disorders; born in foreign countries; hospitalised for dental reasons; edentulous for age; and not accompanied by at least one parent.

Before enrolment, the parents agreed to participate and signed an informed consent form.

Subsequently, the study population was divided into two groups based on age: Group A (0–5 years) and Group B (6–12 years).

### 2.3. Study Variables

(1) ECOHIS questionnaire (administered to Group A): 13 items divided into two sections, the Child Impact section and the Family Impact section (Table S3). The Child Impact section includes four domains: Child Symptoms, Child Function, Child Psychology, and Social Interaction. The Family Impact section encompasses two domains: Parental Distress and Family Function. Responses to the ECOHIS are coded as follows: 0 = never; 1 = hardly ever; 2 = occasionally; 3 = often; 4 = very often; and 5 = don’t know. Scores are calculated by summing the response codes for both sections (excluding the response “don’t know”). The child and family impact sections scores range from 0 to 36 and 0 to 16, respectively, while the total score ranges from 0 to 52. A higher ECOHIS score indicates a greater impact on and poorer oral health-related quality of life [14].

(2) P-CPQ questionnaire (administered to Group B): 16 items, in its validated short version, to assess children’s oral health and its effect on their overall well-being across four domains: Oral Symptoms, Functional Limitations, Emotional Well-being, and Social Well-being (Table S2). Responses are rated using a five-point Likert scale, with values assigned as follows: don’t know = 0, never = 1, once or twice = 2, sometimes = 3, often = 4, and every day or almost every day = 5. The total score for the P-CPQ ranges from 0 to 80, with higher scores indicating a poorer quality of life.

(3) FIS questionnaire: 8 items addressing the impact of oral health on families’ quality of life, divided into three domains: Family Activities, Parental Emotions, and Family Conflicts (Table S3). The FIS total score ranges from 0 to 40, with response options and scoring consistent with the P-CPQ; higher scores indicate poorer quality of life.

(4) Parents’ rating of their children’s oral health: as part of a comprehensive assessment, and in line with previous studies, parents provided ratings of their children’s oral health through two key questions [15]. First, they rated their child’s oral health status by answering the question “How would you classify your child’s oral health?”. The responses were coded on the following 5-point scale: 1 = poor, 2 = below average, 3 = average, 4 = above average, 5 = excellent. Subsequently, parents evaluated how their child’s oral health affected their general well-being using another 5-point Likert scale: 1 = not at all, 2 = a little, 3 = moderately, 4 = significantly, and 5 = very significantly (Table S4).

(5) Socio-demographic data, including parents’ nationality (categorised as EU or non-EU) and education level (none, elementary school, middle school, high school diploma, university degree), were also collected (Table S5). Additionally, the age at which each child had their first dental visit was recorded.

(6) Clinical oral examination. Easy, reproducible, and validated standardised epidemiological indices from the literature were used to assess the oral health status of the recruited subjects: Decayed–Missing–Filled Teeth Index (dmft/DMFT), Index of Orthodontic Treatment Need (IOTN), and Modified Gingival Index (MGI). All examinations were conducted by three dental practitioners.

The dmft/DMFT (decayed, missing, and filled teeth) index is commonly used to evaluate oral health status by quantifying the extent of dental caries, tooth loss, and restorations in both primary (dmft) and permanent (DMFT) dentition. In mixed dentition, the two scores are summed to obtain a combined dmft/DMFT value [16]. The total score documents the overall number of decayed, missing, and filled teeth, reflecting an individual’s caries experience [17,18]. Based on this score, patients were categorised into two groups: no caries experience (score = 0) and caries experience (score ≥ 1). To evaluate the mean caries experience across our sample, the Average Caries Index (ACI) was calculated using dmft//DMFT scores for all three developmental dentitions: deciduous, mixed, and permanent.

The IOTN assesses orthodontic treatment needs, focusing on clinical factors. It evaluates the severity of malocclusions that may lead to functional problems, such as crossbite, open bite, and excessive overjet. It is scored as follows: grade 1 = minimal malocclusion, grade 2 = mild, grade 3 = moderate, grade 4 = severe, and grade 5 = very severe malocclusion [19]. The IOTN was analysed in terms of patients without need for treatment (=1–2), borderline cases (=3), and patients with need for treatment (=4–5). Non-collaborating patients for whom the index could not be recorded were excluded from the statistical analysis. For children in Group A (aged < 6 years), the index was employed as a screening tool rather than a diagnostic measure to identify potential malocclusions.

The Modified Gingival Index (MGI) assesses the severity of gingival inflammation. This index employs a non-invasive method of visual examination without the need for probing that could induce bleeding. It is sensitive in detecting early stages of gingivitis and is widely used in clinical trials. The scores are based on the following criteria: score 0 = normal (no inflammation), score 1 = mild inflammation (slight colour change, minor texture change), score 2 = mild inflammation affecting the entire gingival unit, score 3 = moderate inflammation (redness, swelling, oedema, or hypertrophy), and score 4 = severe inflammation (marked redness, oedema, hypertrophy, spontaneous bleeding, or ulceration). The scores for each tooth are recorded, and the overall gingival health is assessed based on the average score [20]. The MGI was categorised into three groups: healthy (=0), mild inflammation (=1–2), and severe inflammation (=3–4).

### 2.4. Statistical Analysis

The two age groups (Group A: 0–5 years; Group B: 6–12 years) were analysed separately.

The obtained data were evaluated separately for each questionnaire, and subsets and total scores were analysed in relation to the intraoral indices recorded during the clinical examination.

Subsequently, a comparison between the two groups was performed.

Finally, the parents’ backgrounds, comprising their country of origin and education, were correlated with the results to investigate possible associations.

Descriptive statistics were reported as frequencies and percentages for qualitative data, while continuous variables were presented as means ± standard deviation (SD) or medians with interquartile ranges (IQRs).

Differences in the frequencies of each variable were evaluated using the chi-square test or Fisher’s exact test, when appropriate. Wilcoxon–Mann–Whitney’s test was used to compare the median.

All statistical tests were two-sided, and a *p*-value < 0.05 was considered significant. All analyses were performed using the statistical package Stata (version 18.0, Stata Corporation, College Station, TX, USA).

## 3. Results

### 3.1. Sample Analysed

The final study population consisted of 131 children aged 0–12 years, including 54 females and 77 males. The sample was stratified into two age groups. Group A (0–5 years) comprised 60 children (21 females and 39 males), with a mean age of 3.18 years (range: 0.92–5.96, STD: ±1.58). Group B (6–12 years) included 71 children (33 females and 38 males), with a mean age of 9.85 years (range: 6.01–12.97, STD: ±2.07).

The parents of all 131 patients completed the questionnaires. Most were of European origin (88%), and nearly half (49.4%) had completed secondary education. Additionally, approximately one-third of the mothers held a university degree, while only 13.6% of the fathers did (Table 1).

According to age, the ECOHIS questionnaire was administered to Group A (n = 60), while the short version-PCPQ, associated with the Family Impact Scale (FIS), was administered to Group B (N = 71).

As shown in Table 2, the scores were generally low compared to the maximum possible values for each questionnaire, indicating a limited perceived impact of oral health on the quality of life in the study population.

### 3.2. Oral Health Conditions

Caries Experience (dmft/DMFT)

**Figure 1:**
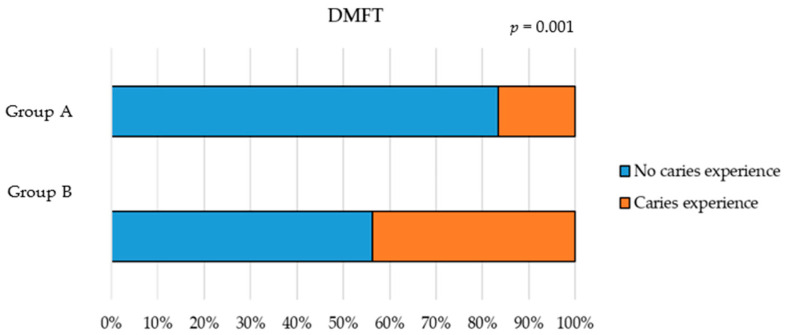
dmft/DMFT index comparison between Group A (0–5 years) and Group B (6–12 years).

From the assessment of caries experience through the dmft/DMFT index, it emerged that 83.3% of the children in Group A (0–5 years) had no experience of caries. The condition worsened in Group B (6–12 years), with 43.7% having experienced caries (*p* = 0.001) (Figure 1).

In Group A, a significant association was found between caries experience and the ECOHIS score. Children with caries had notably higher total ECOHIS scores than caries-free children (mean 12.1 vs. 3.8; *p* = 0.011). Although the differences in the child impact domain were not statistically significant, the family impact domain showed a marked increase among those with caries experience (mean 1.3 vs. 5.1, *p* = 0.008).

In older children (Group B), the number of decayed, missing, or filled teeth increased; however, no statistically significant associations were found between the dmft/DMFT and PCPQ scores. Conversely, although the total FIS score did not reach statistical significance, a significant difference was observed only in the Family Conflict domain (*p* = 0.047) (Table 3).

The Average Caries Index was analysed in both groups (Table 4). In Group A (<6 years), no variations between deciduous and mixed dentition emerged; however, when considering only DMFT, it became 0, confirming an exclusive primary dentition profile. In Group B, the elevated dmft/DMFT (1.9 ± 3.2) compared to dmft alone (1.6 ± 2.9) indicates emerging permanent tooth caries alongside persistent primary tooth caries. The high standard deviation suggests a significant variability in both groups.

Orthodontic Treatment Need (IOTN)

In Group A, the IOTN was recorded in 26 children, while 34 children could not be assessed due to a lack of compliance. Among those evaluated, 20 children (76.9%) showed normal occlusion, 3 (11.5%) were classified as borderline cases, and 3 (11.5%) showed potential malocclusion.

Among Group B, a greater proportion of children demonstrated borderline (14.8%) or definite orthodontic treatment needs (22.2%).

**Figure 2:**
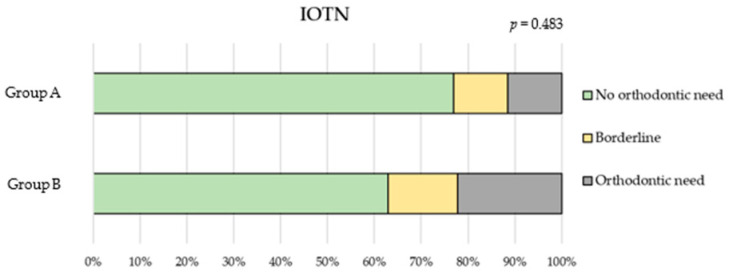
IOTN index comparison between Group A (0–5 years) and Group B (6–12 years).

However, there were no statistically significant differences between the groups (Figure 2) or questionnaire scores according to orthodontic treatment need (Table 5).

Gingival Inflammation Status (MGI)

The gingival inflammation status, measured using the MGI Index, showed that in Group A, 50% of the subjects were healthy and 50% had mild inflammation.

**Figure 3:**
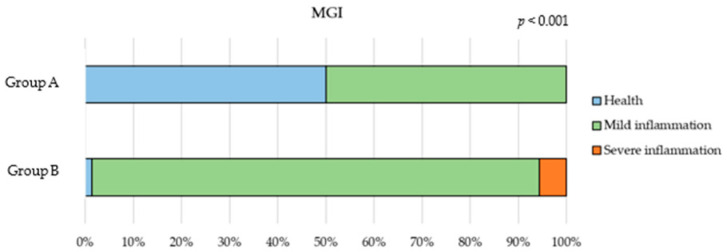
MGI index comparison between Group A (0–5 years) and Group B (6–12 years).

In contrast to Group A, inflammation significantly increased in Group B (*p* < 0.001), with 94.3% and 5.7% of patients showing mild and severe inflammation, respectively (Figure 3). The only patient with healthy gums (a 12-year-old child with type 1 neurofibromatosis) was excluded from the statistical analysis to maintain homogeneity and avoid confounding.

In Group A, the ECOHIS questionnaire revealed no statistically significant differences in oral health impact between children with healthy gums (n = 30) and those with mild inflammation (n = 30) in any of the domains.

The mean scores in the Child Impact domain were low in both groups (3.6 vs. 3) (Table 6). The mean total ECOHIS scores were similar, regardless of the presence of inflammation.

In Group B, the PCPQ and FIS questionnaires demonstrated marked disparities between patients with mild (n = 66) and severe inflammation (n = 4). The total PCPQ mean score was 6.3, and there was a significant difference between patients with mild and severe inflammation (5.6 vs. 17.5, *p* = 0.004). In addition, the Functional Limitations and Emotional Well-being scores were significantly different between the two groups (*p* = 0.009). No FIS domain reached statistical significance; however, the total FIS score increased in the severe inflammation group (Table 6).

**Figure 4:**
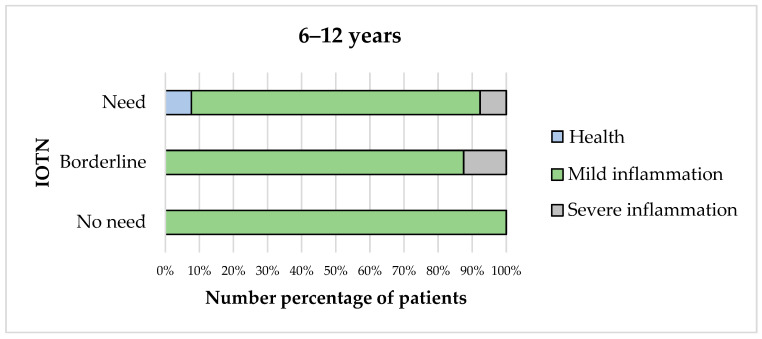
Association between MGI and IOTN in Group B (6–12 years).

When comparing the MGI and IOTN scores, statistical analysis revealed no significant association in Group B (6–12 years) (Figure 4). However, severe inflammation occurred among children with borderline or definite treatment needs (*p* = 0.138).

### 3.3. Supplementary Questions

**Figure 5:**
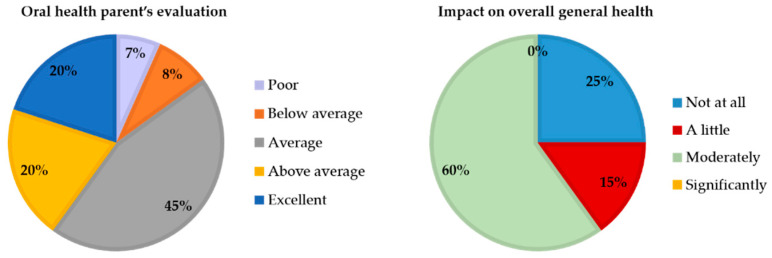
Distribution of supplementary question results in Group A (0–5 years).

In Group A, 85% of parents rated their child’s oral health from “good/average” to “excellent”. Over half of the parents (60%) reported that oral health had only a moderate impact on their child’s overall well-being (Figure 5).

**Figure 6:**
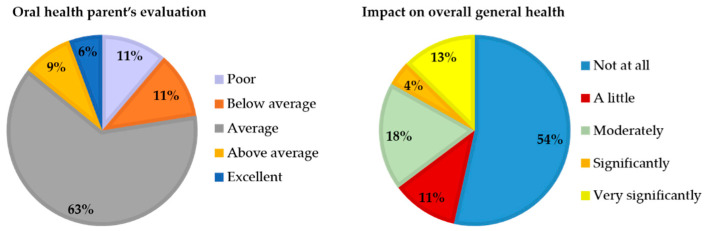
Distribution of supplementary question results in Group B (6–12 years).

In Group B, 63% of parents assessed their child’s oral cavity status as “average,” while 15% considered it “above average” or “excellent”, and 22% rated it “below average” or “poor”. Based on the responses, oral health did not appear to substantially influence the child’s overall well-being, except in 17% of cases, where the impact was reported as ranging from “Significantly” to “Very significantly” (Figure 6).

A comparison across age groups showed a decline in the assessment of children’s oral health and a reduced perceived impact on overall well-being with increasing age.

When analysing the responses to the supplementary questions in association with ECOHIS and PCPQ/FIS, a significant difference emerged: parental judgment appeared to be influenced by caries experience in Group A (*p* = 0.008; Table 7) and by gingival health in Group B (*p* < 0.001; Table 8).

### 3.4. Sociodemographic Background

When comparing dmft/DMFT with parental nationality, 92% of patients without caries experience had EU mothers (*p* = 0.021). Similar results were obtained for fathers (Table 9).

Furthermore, a significant association was found between parental educational attainment and oral health indices. Lower educational levels of either the mother or father were correlated with higher caries experience scores (*p* = 0.036; Table 10) and poorer gingival health (*p* = 0.041; Table 11).

### 3.5. Age at First Dental Examination

**Figure 7:**
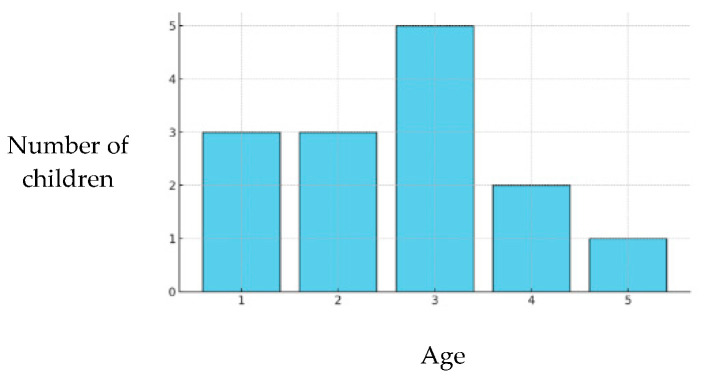
Age at first dental visit in Group A (0–5 years).

The descriptive analysis of the age at the first dental visit in Group A revealed a mean value of 2.64 (±1.22), with both the median and mode equal to 3. The observed range was 1–5. The distribution showed that most children had their first dental visit between the ages of 2 and 3 years, with a peak at 3 years, which appears to be the most common age for the first dental consultation. Visits at 4 and 5 years of age were less frequent, and visits at 1 year were present but were relatively uncommon (Figure 7).

A significant proportion of children (77%) had never undergone dental visits by the age of 5 years.

**Figure 8:**
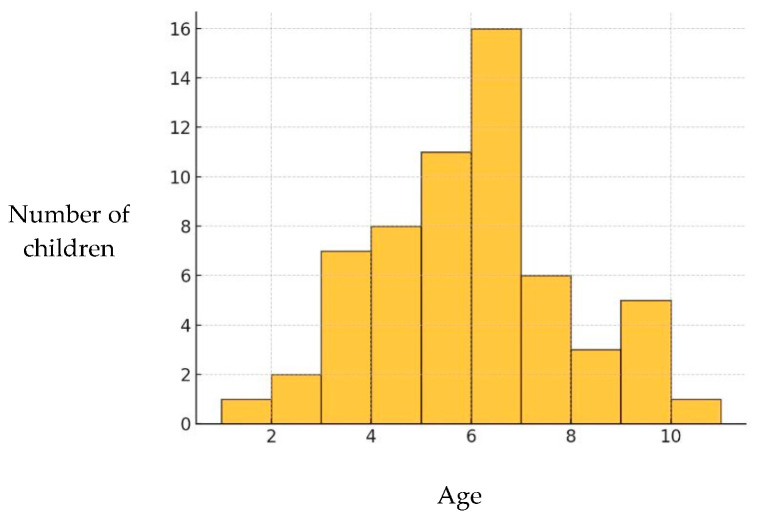
Age at first dental visit in Group B (6–12 years).

The analysis in Group B revealed a mean value of 5.5 (±1.96) years, with both the median and mode being 6.0. The minimum recorded age was 1 year, while the maximum was 10 years, reflecting a wide range in the timing of the first dental visit. A pronounced peak was observed between 5 and 6 years of age, which was the most common age for initial access to dental care. Extreme values corresponding to very young (1–2 years) and older ages (10–11 years) were rare, indicating a lower incidence of first dental visits in these age groups (Figure 8). Notably, 8 (11%) children had never undergone a dental visit by the age of 12 years.

When caries prevalence was correlated with the timing of the first dental visit, significant differences emerged in Groups A and B (Table 12). Children with caries experience tended to have a later first dental visit than those who remained caries-free.

## 4. Discussion

This study aimed to assess the impact of children’s oral health on their own quality of life and that of their families, highlighting the broader psychosocial implications of oral health conditions in the paediatric population.

Previous studies have shown that family plays a crucial role in shaping a child’s oral health, as parental knowledge, attitudes, and behaviours can significantly influence a child’s oral hygiene practices and overall dental care [21]. Analyses of the collected data in the present study confirmed that socioeconomic status is a key factor in determining children’s oral health outcomes. Families with lower educational levels or income often face more difficulties accessing preventive dental care, leading to higher rates of oral diseases [22,23,24]. In this study, non-European origin and lower parental education levels were associated with worse oral health conditions, although these differences were not always statistically significant.

The prevalence of dental caries observed in our study, 16.7% in Group A (0–5 years) and 43.7% in Group B (6–12 years), is consistent with the data present in the literature, where the estimated prevalence is 21.6% at 4 years of age [25] and 43.1% at 12 years [26]. Our findings are slightly more favourable for Group A, which may be attributed to two main factors: our younger cohort includes children below the age of 4, who are typically at lower risk for caries, and over the 17 years since the publication of the Italian Ministry of Health’s guidelines for the prevention of oral diseases in children and adolescents [20], preventive measures may have had the desired effect in reducing the overall incidence of dental caries. More recent data for children aged 0–5 years reported a lower overall caries prevalence of 8.2% across the entire age range [27]. However, among children aged 4–5.9 years, the prevalence rose to 14.7%, which is closer to the findings of our study. This difference may be explained by the variations in the age distribution of the children assessed and the relatively small sample size in our cohort. However, it is worth noting that the above-mentioned prevalence, reported by Colombo et al. (2019) [27], was based on an online questionnaire administered to parents, which may introduce reporting bias and limit comparability with the data obtained through clinical examination.

Regarding the 6–12 years age group, updated data from the 2020 Italian National Pathfinder survey on 12-year-old children showed that only 30.5% were caries-free [28], resulting in a caries prevalence of 69.5%. However, this study used a different caries detection method, namely ICDAS, instead of the traditional DMFT. Unlike the DMFT, the ICDAS also records early enamel demineralisations. This could explain the higher caries prevalence observed by Campus et al.

Our findings indicate that younger children generally exhibit acceptable oral health; however, the presence of caries is associated with a significant decline in both the child’s and family’s quality of life. Children with caries had significantly higher total ECOHIS scores than those without, with a particularly marked increase in the family impact domain. These results suggest that caries experience significantly damages oral health-related quality of life (OHRQoL) in early childhood, particularly in terms of family burden. The ECOHIS questionnaire effectively captured significant OHRQoL deterioration in young children with caries, particularly in the family impact and total scores (*p* < 0.05). In the older age group, caries prevalence increased, although no significant association was found between the dmft/DMFT and PCPQ scores. Nonetheless, a significant difference emerged in the Family Impact Scale (FIS) domain, suggesting that caries in older children may contribute to greater familial stress, even when the direct impact on the child’s perceived well-being is less evident. The comparison between questionnaire scores and dmft/DMFT values in this study supports the existing literature, further strengthening the link between oral health status and quality of life [10,11,14,29].

The prevalence of IOTN grades 4–5 observed in our sample was 11.5% in Group A (0–5 years) and 22.2% in Group B (6–12 years), representing potential risk and definite high orthodontic treatment need, respectively. These values are lower than those reported previously. A 2020 study by Grippaudo et al. found a prevalence of 23.7% in children aged 2–7 years and 38.1% in those aged 8–13 years [30]. This discrepancy may be partly explained by the slightly different age ranges considered, but more importantly, by the use of different assessment indices. While our study employed the IOTN, a widely used international index, Grippaudo et al. [30] used the ROMA and Baby-ROMA indices, which are applied exclusively in the Italian context.

When comparing the ECOHIS questionnaire to the IOTN, no clear association emerged between the IOTN grades and ECOHIS scores. Interestingly, higher ECOHIS scores were sometimes observed in children with lower grades. This suggests that, in early childhood, perceptions of OHRQoL are more influenced by general oral health conditions, specifically caries experience, than by malocclusion severity.

In Group B (6–12 years), both the PCPQ and FIS scores tended to increase with greater orthodontic need, reflecting a closer alignment between clinical orthodontic status, subjective quality of life measures, and parental perception. Although these differences did not reach statistical significance, the trend aligned with previous studies, highlighting the psychosocial impact of malocclusion during middle childhood and early adolescence [31,32].

The literature data on the prevalence of gingivitis in the paediatric population in Italy are limited and not easily accessible. Campus et al. (2007) reported that 52.5% of a sample of 12-year-old Italian children presented with gingival health issues, including 23.8% with gingival bleeding and 28.7% with calculus [26]. Reliable data on younger children are lacking. In our study, the prevalence in Group A (0–5 years) showed a balanced distribution, with 50% of the children presenting with healthy gingiva and 50% presenting with mild gingivitis. In contrast, Group B (6–12 years) showed concerning results, with 94.3% showing mild gingivitis, 5.7% severe gingivitis, and only one child with healthy gingiva.

When the questionnaire scores and MGI were compared, the PCPQ detected significant OHRQoL deterioration in older children (Group B) with severe gingival inflammation. The PCPQ and FIS questionnaires demonstrated marked disparities between those with mild and severe inflammation (4 vs. 12.5, *p* = 0.004), demonstrating that the severity of gingival inflammation has a measurable impact on both the child’s perceived oral health–related quality of life and the family’s daily functioning. These findings highlight the importance of early diagnosis and management of gingival conditions, especially in older children, to prevent not only clinical progression but also psychosocial consequences for both children and parents. Additionally, the Functional Limitations and Emotional Well-being scores differed significantly between the two groups. No FIS domain reached statistical significance; however, the total FIS score increased in the severe inflammation group, suggesting a greater family impact.

The ECOHIS scores remained uniformly low and insensitive to gingival status differences in younger children, suggesting minimal disruption to the child’s quality of life. This is partially consistent with clinical observations, where approximately 50% of the children had healthy gums and the other 50% had mild gingival inflammation.

As noted by Pahel et al. (2007), the relatively mild nature of gingival issues in this age group likely explains why the ECOHIS failed to detect significant differences in OHRQoL, as their impact on daily functioning and family well-being was minimal [11]. However, the low scores may reflect either parental underestimation of the oral health impact in very young children or genuinely milder psychosocial consequences associated with early-stage gingival inflammation. In contrast, the PCPQ’s ability to differentiate more clearly in older children highlights its usefulness in assessing the burden of more advanced oral diseases [8].

Overall, the questionnaire scores were relatively low compared to the maximum possible values, which may suggest a weak correlation between parental perception and a child’s actual oral health status. Additionally, confounding factors may have led parents to focus more on other coexisting health or social issues, diverting their attention away from dental problems.

Nevertheless, the worsening of all clinical indices (MGI, DMFT, and IOTN) with increasing age indicates the urgent need for early preventive interventions.

In this regard, the analysis of the age at the first dental examination was concerning. Despite recommendations from institutional guidelines (e.g., American Association of Paediatric Dentistry, Italian Ministry of Health) to schedule the first dental visit within the first 24 months of life [33,34], these guidelines are seldom followed. The average age at the first dental examination was 6 years, with the majority of children in the 0–5 years age Group A (Group A) never having been seen by a dentist. Moreover, in Groups A and B, caries-free children had their first dental visit significantly earlier than those with caries. The statistically significant differences observed across both cohorts reinforce the role of early dental attendance as a cornerstone of caries prevention.

This finding suggests a potential gap in knowledge and understanding of the importance of early dental visits and prevention, highlighting the need to raise awareness of the importance of early dental care. Establishing a dental home by age one and providing follow-up care at developmental milestones should therefore be viewed not only as the best practice for individual patient care but also as a population-level strategy to meet international caries reduction goals, as defined by the WHO [1].

The WHO Global Oral Health Action Plan envisions that by 2030, countries will adopt measurable targets for paediatric oral health, with particular attention to dental caries and malocclusion. Planned actions to achieve these targets include promoting oral health education, reducing free sugar intake, and ensuring equitable access to preventive and therapeutic dental services for all children [1].

A limitation of this study is its small sample size, which may limit the generalisability of the findings. Furthermore, reliance on self-reported data, despite the use of validated questionnaires, introduces potential bias related to subjective reporting. Further research involving larger and more diverse cohorts is essential to validate these findings and explore the influence of socioeconomic, cultural, and behavioural factors in greater depth.

## 5. Conclusions

The findings of this study demonstrate that oral diseases have a tangible impact on children’s quality of life and contribute to family stress, particularly in socioeconomically disadvantaged contexts. In younger children (0–5 years), the presence of caries was significantly associated with higher ECOHIS scores, especially in the “Family Impact” domain, underscoring the burden that oral health issues place on the family’s well-being. In older children (6–12 years), although no strong association emerged between caries and PCPQ scores, family conflict (FIS) increased, and the quality of life declined in cases of severe gingival inflammation.

Considering the observed increase in PCPQ scores with both IOTN and age, it would be worthwhile to design future studies specifically targeting adolescents older than 12 years of age. Such research could provide valuable insights into whether the psychosocial impact of malocclusion and the importance attributed to dental aesthetics become more pronounced with advancing age during adolescence.

The progressive worsening of clinical indices (DMFT, MGI, and IOTN) with age, coupled with the average age at the first dental visit being well beyond the recommended 24 months, highlights a systematic delay in preventive care with long-term implications. These delays, along with differences observed by parental nationality and education, highlight the need for targeted interventions to reduce disparities and promote timely access to dental services.

In this regard, paediatricians, who see children from birth, are well placed to encourage early dental visits and refer families within the recommended timeframe. According to the WHO global strategy, family-centred education and public health strategies focusing on early prevention, such as promoting tooth brushing with fluoride toothpaste from the eruption of the first tooth and reducing free sugar intake, should be integrated into national oral health policies.

## Figures and Tables

**Table 1:** Parents’ nationality and level of education.

	Mother	Father	Total
	N (%)	N (%)	N (%)
Nationality		*	
EU	116 (88.5)	114 (88.4)	230 (88.5)
Extra EU	15 (11.5)	15 (11.6)	30 (11.5)
Education level	**	***	
Not going to school	1 (0.8)	0	1 (0.4)
Primary education	0	1 (0.8)	1 (0.4)
Middle school	30 (24.0)	36 (30.5)	66 (27.2)
Secondary education	55 (44.0)	65 (55.1)	120 (49.4)
Postsecondary education	39 (31.2)	16 (13.6)	55 (22.6)

* 2 missing; ** 6 missing; *** 13 missing.

**Table 2:** Distribution of the scores of the ECHOIS, PCPQ, and FIS questionnaires.

	Median (IQR)	Mean ± SD
**ECOHIS_0–5_ scores**		
Total	4 (1–7)	5.2 ± 5.6
Child impact	2 (0–5)	3.3 ± 4.4
Family impact	0 (0–3)	1.9 ± 2.8
**16-PCPQ_6–12_ scores**		
Total	5 (2–8)	6.2 ± 7.4
Oral symptoms	2 (1–5)	2.9 ± 2.4
Functional limitations	0 (0–3)	1.4 ± 2.1
Emotional well-being	0 (0–2)	1.3 ± 2.9
Social well-being	0 (0–0)	0.6 ± 2.1
**FIS_6–12_ scores**		
Total	5 (1–11)	6.7 ± 6.6
Parental emotions	1 (0–3)	1.7 ± 2.0
Parental family activity	3 (0–6)	3.6 ± 3.8
Family conflict	0 (0–3)	1.5 ± 1.9

**Table 3:** Results of ECOHIS_0–5_, 16-PCPQ_6–12_, and FIS_6–12_ mean ± SD scores according to dmft/DMFT domains.

	dmft/DMFT			
	No Caries Experience	Caries Experience	Total	*p*
**ECOHIS_0–5_ scores**				
yeTotal	3.8 ± 3.3	12.1 ± 9.9	5.2 ± 5.8	**0.011**
Child impact	2.5 ± 2.9	7 ± 7.8	3.3 ± 4.4	0.102
Family impact	1.3 ± 1.9	5.1 ± 4.1	1.9 ± 2.8	**0.008**
**16-PCPQ_6–12_ scores**				
Total	5.6 ± 6.1	7.0 ± 8.8	6.2 ± 7.4	0.554
Oral symptoms	2.9 ± 2.5	3 ± 2.4	2.9 ± 2.4	0.759
Functional limitations	1.4 ± 2.0	1.4 ± 2.1	1.4 ± 2.1	0.799
Emotional well-being	1.0 ± 2.3	1.7 ± 3.4	1.3 ± 2.9	0.444
Social well-being	0.3 ± 1.2	0.9 ± 2.9	0.5 ± 2.1	0.366
**FIS_6–12_ scores**				
Total				0.059
Parental emotions	1.4 ± 1.8	2.0 ± 2.2	1.6 ± 2.0	0.161
Parental family activity	2.9 ± 3.2	4.4 ± 4.4	3.6 ± 3.8	0.179
Family conflict	1.0 ± 1.4	2.1 ± 2.2	1.5 ± 1.9	**0.047**

**Table 4:** Average Caries Index in Group A (0–5 years) and Group B (6–12 years).

	ACI (dmft)	ACI (dmft/DMFT)	ACI (DMFT)
Group A	1.1 ± 2.8	1.1 ± 2.8	0 ± 0
Group B	1.6 ± 2.9	1.9 ± 3.2	0.6 ± 1.4

**Table 5:** Results of ECOHIS_0–5_, 16-PCPQ_6–12_, and FIS_6–12_ mean ± SD scores according to IOTN domains.

	IOTN				
	No Need	Borderline	Need	Total	*p*
**ECOHIS_0–5_ scores**					
Total	4.9 ± 5.9	4.3 ± 2.1	0.7 ± 1.2	4.3 ± 5.3	0.213
Child impact	2.1 ± 3.1	0 ± 0	0 ± 0	1.6 ± 2.9	0.598
Family impact	2.8 ± 3.5	4.3 ± 2.1	0.7 ± 1.2	2.7 ± 3	0.247
**16-PCPQ_6–12_ scores**					
Total	5.2 ± 7.4	6.1 ± 4.1	6.3 ± 4.9	5.6 ± 6.4	0.342
Oral symptoms	2.8 ± 2.4	2.6 ± 1.9	3.8 ± 3.2	3.0 ± 2.5	0.623
Functional limitations	1.1 ± 1.9	1.8 ± 2.5	1.3 ± 1.7	1.2 ± 1.9	0.851
Emotional well-being	0.9 ± 2.4	1.3 ± 1.6	1.1 ± 1.7	1.0 ± 2.1	0.439
Social well-being	0.5 ± 2.6	0.5 ± 0.8	0.2 ± 0.4	0.4 ± 2.1	0.406
**8-FIS_6–12_ scores**					
Total	6.5 ± 5.9	10 ± 9.5	5.6 ± 4.8	6.8 ± 6.4	0.562
Parental emotions	1.6 ± 1.8	2.0 ± 2.7	1.1 ± 1.6	1.6 ± 1.9	0.615
Parental family activity	3.5 ± 3.6	4.9 ± 4.9	2.9 ± 3.6	3.6 ± 3.8	0.627
Family conflict	1.4 ± 1.7	3.1 ± 2.5	1.6 ± 2.0	1.7 ± 2.0	0.201

**Table 6:** Results of ECOHIS_0–5_, 16-PCPQ_6–12_, and FIS_6–12_ mean ± SD scores according to MGI domains.

		MGI			
	Healthy Gums	Mild Inflammation	Severe Inflammation	Total	*p*
**ECOHIS_0–5_ scores**					
Total	4.8 ± 5.1	5.5 ± 6.5	-	5.2 ± 5.8	0.853
Child impact	3.6 ±3.8	3 ± 4.9	-	3.3 ± 4.4	0.301
Family impact	1.3 ± 2.1	2.5 ± 3.2	-	1.9 ± 2.8	0.246
**16-PCPQ_6–12_ scores**					
Total	-	5.6 ± 6.7	17.5 ± 11.0	6.3 ± 7.4	**0.004**
Oral symptoms	-	2.8 ± 2.3	5.3 ± 3.0	2.9 ± 2.4	0.103
Functional limitations	-	1.2 ± 1.9	4.8 ± 2.2	1.4 ± 2.1	**0.009**
Emotional well-being	-	1.1 ± 2.5	5.5 ± 5.2	1.3 ± 2.9	**0.009**
Social well-being	-	0.5 ± 2.0	2.0 ± 3.4	0.6 ± 2.1	0.215
**8-FIS_6–12_ scores**					
Total	-	6.5 ± 6.7	10.0 ± 3.4	6.7 ± 6.6	0.123
Parental emotions	-	1.6 ± 2.0	3.0 ± 1.4	1.7 ± 2.0	0.095
Parental family activity	-	3.5 ± 3.9	4.3 ± 1.7	3.5 ± 3.8	0.383
Family conflict	-	1.4 ± 1.8	2.8 ± 2.5	1.5 ± 1.9	0.255

**Table 7:** Correlation between supplementary questions and caries experience in Group A.

	dmft/DMFT			
	No Caries Experience	Caries Experience	Total	
	N (%)	N (%)	N (%)	*p*
GROUP A: Oral health parents’ evaluation				0.008
Poor	1 (2.0)	3 (30.0)	4 (6.7)	
Below Average	3 (6.0)	2 (20.0)	5 (8.3)	
Average	23 (46.0)	4 (40.0)	27 (45.0)	
Above Average	12 (24.0)	0	12 (20.0)	
Excellent	11 (22.0)	1 (10.0)	12 (20.0)	

**Table 8:** Correlation between supplementary questions and gingival inflammation in Group B.

	Mild Inflammation	Severe Inflammation	Total	
	N (%)	N (%)	*p*	
Group B: Oral health parents’ evaluation			*	<0.001
Poor	4 (6.1)	4 (100.0)	8 (11.4)	
Below Average	7 (10.6)	0	7 (10.0)	
Average	45 (68.2)	0	45 (64.3)	
Above Average	6 (9.1)	0	6 (8.6)	
Excellent	4(6.1)	0	4 (5.7)	

* 1 excluded for maintaining statistical homogeneity (only patient with healthy gums).

**Table 9:** Correlation between parents’ nationality and caries experience in Group A (0–5 years).

	dmft/DMFT			
	No Caries Experience	Caries Experience	Total	
Group A: Nationality	N (%)	N (%)	N (%)	*p*
**Mother**				**0.021**
EU	46 (92.0)	6 (60.0)	52 (86.7)	
Extra EU	4 (8.0)	4 (40.0)	8 (13.3)	
**Father** *				**0.014**
EU	43 (87.9)	5 (50.0)	48 (81.4)	
Extra EU	6 (12.2)	5 (50.0)	11 (18.6)	

* 1 missing.

**Table 10:** Correlation between mothers’ education and caries experience in Group B (6–12 years).

	dmft/DMFT			
	No Caries Experience	Caries Experience	Total	
	N (%)	N (%)	N (%)	*p*
Group B: *Mother’s education*			*	0.036
None or Primary school	0	1 (3.5)	1 (1.5)	
Middle school	5 (12.8)	11 (37.9)	16 (23.5)	
High School	20 (51.3)	11 (37.9)	31 (45.6)	
Degree	14 (35.9)	6 (20.7)	20 (29.4)	

* 1 missing.

**Table 11:** Correlation between fathers’ education and gingival inflammation in Group B (6–12 years).

	MGI			
	Mild Inflammation	Severe Inflammation	Total	
	N (%)	N (%)	N (%)	*p*
Group B: *Father’s education*			*	0.041
Primary school	1 (1.7)	0	1 (1.6)	
Middle school	17 (28.3)	4 (100.0)	21 (32.8)	
High School	33 (55.0)	0	33 (51.6)	
Degree	9 (19.0)	0	9 (14.1)	

* 7 missing.

**Table 12:** Correlation between caries experience and age at first dental visit in Groups A (0–5 years) and B (6–12 years).

Age		No Caries Experience	Caries Experience	*p*
Group A	Median (IQR) Mean ± SD	2 (1.0–3.0) 2.1 ± 1.3	3.5 (3–5) 3.8 ± 1.5	**0.002** **0.002**
Group B	Median (IQR) Mean ± SD	5 (4.0–6.0) 5.2 ± 2.0	6.0 (5.0–8.0) 6.3 ± 2.2	**0.013** **0.018**

## Data Availability

The datasets generated and/or analysed during the current study are not publicly available as they are being utilised for ongoing purposes; however, they can be made available by the corresponding author upon reasonable request.

## Supplementary Materials

The following supporting information can be downloaded at: https://www.mdpi.com/article/10.3390/children12091119/s1, Table S1: ECOHIS Items; Table S2: 16-PCPQ Items; Table S3: 8-FIS Items; Table S4: Supplementary questions; Table S5: Sociodemographic family background.

## Abbreviations

The following abbreviations are used in this manuscript.

IRCCS G. Gaslini	Istituto di Ricerca e Cura a Carattere Scientifico Giannina Gaslini
QoL	Quality of Life
WHO	World Health Organisation
OHRQoL	Oral Health-Related Quality of Life
COHQoL	Child Oral-Health-Related Quality of Life
COHIP	Child Oral Health Impact Profile
Child-OIDPs	Child Oral Impacts on Daily Performances
CPQ	Child Perceptions Questionnaire
ECOHIS	Early Childhood Oral Health Impact Scale
PCPQ	Parental–Caregiver Perceptions Questionnaire
FIS	Family Impact Scale
DMFT	Decayed (D), Missing (M), and Filled (F) for Permanent Teeth
dmft	Decayed (D), Missing (M), and Filled (F) for primary teeth
IOTN	Index of Orthodontic treatment Need
MGI	Modified Gingival Index
EU	European Union
years	years

## References

[1] World Health Organization (2025). Global Strategy and Action Plan on Oral Health 2023–2030.

[2] Locker D., Jokovic A., Stephens M., Kenny D., Tompson B., Guyatt G. (2002). Family Impact of Child Oral and Oro-Facial Conditions. Community Dent. Oral. Epidemiol..

[3] Quadri M.F.A., Jaafari F.R.M., Mathmi N.A.A., Huraysi N.H.F., Nayeem M., Jessani A., Tadakamadla S.K., Tadakamadla J. (2021). Impact of the Poor Oral Health Status of Children on Their Families: An Analytical Cross-Sectional Study. Children.

[4] Lione R., Ralli M., De Razza F.C., D’Amato G., Arcangeli A., Carbone L., Cozza P. (2024). Oral Health Epidemiological Investigation in an Urban Homeless Population. Dent. J..

[5] World Health Organization (2021). Oral Health Country Profile: Italy.

[6] World Health Organization (1995). Quality of Life Assessment: WHOQOL.

[7] Thomson W.M., Foster Page L.A., Malden P.E., Gaynor W.N., Nordin N. (2014). Comparison of the ECOHIS and Short-Form P-CPQ and FIS Scales. Health Qual. Life Outcomes.

[8] Thomson W.M., Foster Page L.A., Gaynor W.N., Malden P.E. (2013). Short-Form Versions of the Parental-Caregivers Perceptions Questionnaire and the Family Impact Scale. Community Dent. Oral. Epidemiol..

[9] Rincón T., Gómez-Polo C., Montero J., Curto D., Curto A. (2025). An Assessment of Oral-Health-Related Quality of Life and Anxiety in Early Adolescents (11–14 Years) at Their First Dental Visit: A Cross-Sectional Study. Children.

[10] Çınar B.K., Bucci R., D’Antò V., Cascella S., Rongo R., Valletta R. (2025). Parental Perceptions and Family Impact on Adolescents’ Oral Health-Related Quality of Life in Relation to the Severity of Malocclusion and Caries Status. Children.

[11] Pahel B.T., Rozier R.G., Slade G.D. (2007). Parental Perceptions of Children’s Oral Health: The Early Childhood Oral Health Impact Scale (ECOHIS). Health Qual. Life Outcomes.

[12] Contaldo M., Della Vella F., Raimondo E., Minervini G., Buljubasic M., Ogodescu A., Sinescu C., Serpico R. (2020). Early Childhood Oral Health Impact Scale (ECOHIS): Literature Review and Italian Validation. Int. J. Dent. Hyg..

[13] Minervini G., Franco R., Marrapodi M.M., Di Blasio M., Ronsivalle V., Cicciù M. (2023). Children Oral Health and Parents Education Status: A Cross Sectional Study. BMC Oral Health.

[14] Li M.Y., Zhi Q.H., Zhou Y., Qiu R.M., Lin H.C. (2015). Impact of Early Childhood Caries on Oral Health-Related Quality of Life of Preschool Children. Eur. J. Paediatr. Dent..

[15] Salerno C., Campus G., Bontà G., Vilbi G., Conti G., Cagetti M.G. (2025). Oral Health-Related Quality of Life in Children and Adolescent with Autism Spectrum Disorders and Neurotypical Peers: A Nested Case–Control Questionnaire Survey. Eur. Arch. Paediatr. Dent..

[16] Ahmad F.N., Moutam N., Sowmya K., Parvatham B.B., Rajesh D., Kumar S. (2022). Evaluation of DMFT in School Going Children of Mixed Dentition Stage: An Original Research. Int. J. Health Sci..

[17] Petersen P.E., Baez R.J., World Health Organization (2013). Oral Health Surveys: Basic Methods.

[18] Klein H., Palmer C.E., Knutson J.W. (1938). Studies on Dental Caries: I. Dental Status and Dental Needs of Elementary School Children. Public Health Rep. (1896–1970).

[19] Brook P.H., Shaw W.C. (1989). The Development of an Index of Orthodontic Treatment Priority. Eur. J. Orthod..

[20] Lobene R.R., Weatherford T., Ross N.M., Lamm R.A., Menaker L. (1986). A Modified Gingival Index for Use in Clinical Trials. Clin. Prev. Dent..

[21] Bilal S., Abdulla A.M., Andiesta N.S., Babar M.G., Pau A. (2021). Role of Family Functioning and Health-Related Quality of Life in Pre-School Children with Dental Caries: A Cross-Sectional Study. Health Qual. Life Outcomes.

[22] Dettori M., Arghittu A., Cappai A., Castiglia P., Campus G., Children’s Smiles Sardinian Group (2024). Impact of Socioeconomic Inequalities on Dental Caries Status in Sardinian Children. Children.

[23] Theristopoulos A., Agouropoulos A., Seremidi K., Gizani S., Papaioannou W. The Effect of Socio-Economic Status on Children’s Dental Health. https://www.jocpd.com/articles/10.22514/jocpd.2024.078.

[24] Vallejos D., Coll I., López-Safont N. (2025). Association Between the Oral Health Status and Sociodemographic Factors Among 5–15-Year-Old Schoolchildren from Mallorca, Spain—A Cross-Sectional Study. Children.

[25] Campus G., Solinas G., Strohmenger L., Cagetti M.G., Senna A., Minelli L., Majori S., Montagna M.T., Reali D., Castiglia P. (2009). National Pathfinder Survey on Children’s Oral Health in Italy: Pattern and Severity of Caries Disease in 4-Year-Olds. Caries Res..

[26] Campus G., Solinas G., Cagetti M.G., Senna A., Minelli L., Majori S., Montagna M.T., Reali D., Castiglia P., Strohmenger L. (2007). National Pathfinder Survey of 12-Year-Old Children’s Oral Health in Italy. Caries Res..

[27] Colombo S., Gallus S., Beretta M., Lugo A., Scaglioni S., Colombo P., Paglia M., Gatto R., Marzo G., Caruso S. (2019). Prevalence and Determinants of Early Childhood Caries in Italy. Eur. J. Paediatr. Dent..

[28] Campus G., Cocco F., Strohmenger L., Cagetti M.G. (2020). Caries Severity and Socioeconomic Inequalities in a Nationwide Setting: Data from the Italian National Pathfinder in 12-Years Children. Sci. Rep..

[29] Anderson H.K., Drummond B.K., Thomson W.M. (2004). Changes in Aspects of Children’s Oral-Health-Related Quality of Life Following Dental Treatment under General Anaesthesia. Int. J. Paediatr. Dent..

[30] Grippaudo C., Quinzi V., Manai A., Paolantonio E.G., Valente F., La Torre G., Marzo G. (2020). Orthodontic Treatment Need and Timing: Assessment of Evolutive Malocclusion Conditions and Associated Risk Factors. Eur. J. Paediatr. Dent..

[31] The Psychosocial Impact of Oral Conditions during Transition to Secondary Education—PubMed. https://pubmed.ncbi.nlm.nih.gov/20073542/.

[32] Locker D., Jokovic A., Tompson B., Prakash P. (2007). Is the Child Perceptions Questionnaire for 11-14 Year Olds Sensitive to Clinical and Self-Perceived Variations in Orthodontic Status?. Community Dent. Oral Epidemiol..

[33] Ministero della Salute Italiano Linee Guida Nazionali per La Promozione Della Salute Orale e La Prevenzione Delle Patologie Orali in Età Evolutiva 2014. https://www.salute.gov.it/new/sites/default/files/imported/C_17_pubblicazioni_2073_allegato.pdf.

[34] (2016). Guideline on Perinatal and Infant Oral Health Care. Pediatr. Dent..

