# Supplementary material for: Parental Perceptions and Actual Oral Health Status of Children in an Italian Paediatric Population in 2024: Findings from an Observational Study
Source: Children (Basel). 2025 Aug 25;12(9):1119. doi: 10.3390/children12091119 (PMC12468668; doi:10.3390/children12091119)
Supplement: Supplementary file 1 [file children-12-01119-s001.zip › children-3793445-supplementary.pdf]

Table S1. ECOHIS ITEMS

How often has your child (had/been)… due to their teeth/mouth?	
Pain in teeth/mouth/jaws	Child Impact
Difficulty drinking hot/cold drinks	
Trouble eating certain foods	
Difficulty pronouncing any words	
Missed daycare, kindergarten, or school	
Irritable/frustrated	
Trouble sleeping	
Avoided smiling or laughing	
Avoided talking	
Have you or someone else in the family…due to problems with your child's teeth or dental treatments	
Been upset	Family Impact
Felt guilty	
Missed work	
Had a financial impact	
Italian Version	
Quante volte il/la suo/a bambino/a … a causa di problemi dentali o di cure odontoiatriche?	
Avverte dolore a denti/ bocca/mascellari	Child Impact
Ha difficoltà a bere bevande calde o fredde	
Ha difficoltà a mangiare alcuni cibi	
Ha difficoltà a pronunciare alcune parole	
Si è assentato/a dalla scuola (asilo nido, ecc.)	
Ha difficoltà a dormire	
Si è irritato o infastidito	
Evita di sorridere o ridere	
Evita di parlare	
Quante volte lei o un suo familiare ... a causa dei problemi dentali o delle cure odontoiatriche del vostro bambino?	
Si è preoccupato/a	Family Impact
Si è sentito/a in colpa	
Si è dovuto/a assentare dal posto di lavoro	
Ha subito una perdita di guadagno	

Table S2. 16- PCPQ ITEMS

During the last 3 months, how often has your child (had/been)… due to their teeth/mouth?	
Bad breath	Oral symptoms
Pain in teeth/mouth	
Food stuck to roof of mouth	
Food caught between teeth	
Difficulty chewing firm foods	Functional Limitations
Breathing through mouth	
Slow eating	

Trouble sleeping	
Irritable/Frustrated	Emotional Well-Being
Upset	
Anxious/Fearful	
Shy/Embarrassed	
Missed school	Social Well-Being
Avoided smiling when around other children	
Had hard time paying attention in school	
Not wanted to talk to other children	

Table S3. 8-FIS ITEMS

During the last 3 months, how often have/has… due to problems with child's teeth or dental treatments?		
You or someone else in the family	Taken time off work	Parental/Family Activity
Your child	Required more attention from you or others in the family	
You or someone else in the family	Had less time for yourself or the family	
You or someone else in the family	Had sleep disrupted	
You or someone else in the family	Been upset	Parental Emotions
You or someone else in the family	Felt guilty	
Your child	Argued with you or others in the family	Family Conflict
Your child	Blamed you or another person in the family	

Table S4. Supplementary questions

How would you rate the health of your child's teeth, lips, jaw, and mouth?
How much has your child's overall well-being been affected by the condition of their teeth, lips, jaw, or mouth?

Table S5. Sociodemographic family background

Interviewed parent
Parent–child relationship
What is the parents' nationality?
What is the parents' level of education?
